# BRISK: Dynamic Encryption Based Cipher for Long Term Security

**DOI:** 10.3390/s21175744

**Published:** 2021-08-26

**Authors:** Ashutosh Dhar Dwivedi

**Affiliations:** Cyber Security Section, Department of Applied Mathematics and Computer Science, Technical University of Denmark, 2800 Kgs. Lyngby, Denmark; adhdw@dtu.dk or ashudhar7@gmail.com

**Keywords:** lightweight cryptography, Internet of Things (IoT), dynamic encryption, block cipher, fast encryption

## Abstract

Several emerging areas like the Internet of Things, sensor networks, healthcare and distributed networks feature resource-constrained devices that share secure and privacy-preserving data to accomplish some goal. The majority of standard cryptographic algorithms do not fit with these constrained devices due to heavy cryptographic components. In this paper, a new block cipher, BRISK, is proposed with a block size of 32-bit. The cipher design is straightforward due to simple round operations, and these operations can be efficiently run in hardware and suitable for software. Another major concept used with this cipher is dynamism during encryption for each session; that is, instead of using the same encryption algorithm, participants use different ciphers for each session. Professor Lars R. Knudsen initially proposed dynamic encryption in 2015, where the sender picks a cipher from a large pool of ciphers to encrypt the data and send it along with the encrypted message. The receiver does not know about the encryption technique used before receiving the cipher along with the message. However, in the proposed algorithm, instead of choosing a new cipher, the process uses the same cipher for each session, but varies the cipher specifications from a given small pool, e.g., the number of rounds, cipher components, etc. Therefore, the dynamism concept is used here in a different way.

## 1. Introduction

Computer devices nowadays are continuously developing, and the performance of these new devices is better than before with powerful resources. Portable devices like tablets and smartphones have replaced basic phones and allow several advanced features similar to a computer. Existing standard cryptographic algorithms were designed to meet the requirements of desktop/server computers. In recent years, the Internet of Things has become popular. These resource-constrained devices are increasingly used in various applications, such as wireless sensor networks and Radio Frequency Identification (RFID). These devices have minimal memory and power, and computing capability. Providing security to these devices with lightweight encryption techniques is a major challenge nowadays. Lightweight cryptography [1] provides a compact encryption algorithm that fits these resource-constrained devices. In this paper, a lightweight block cipher suitable for IoT devices has been developed. A block cipher is the most widely used cryptographic primitive applied to hashing, encryption, random bit generation and message authentication. AES [2] is the standard cryptographic block cipher used widely nowadays. AES has good performance on hardware and software and is generally considered secure after 20 years of cryptanalysis. One obvious question arises for designers, that of why not AES, and indeed, it has been suggested for lightweight encryption. The problem with AES is that it is not a good choice for constrained devices, e.g., in hardware, the area should not exceed 2000 gates, while AES requires 2400 gates. Here, an optimised block cipher BRISK has been proposed that provides excellent performance on software and hardware. To provide the best compatibility with several hardware devices, the encryption algorithm has several ranges of block sizes, and each can fit with a variety of implementations. The algorithm is based on Feistel network Type 3. The Feistel networks are characterised into three types based on the fact that input is divided into two or more sub-words. Secret key plays an essential role in the security of any cipher. Participants using traditional cryptographic algorithms agree on a particular cipher for encryption and decryption and establish a key exchange. An attacker [3,4] is expected to know about the system (specific encryption algorithm), but cannot decrypt the data without the secret key. The secret key changes its values in each communication, but the encryption algorithm remains the same, and therefore the security completely relies on secret keys. According to Kerckhoffs’ principle, a cryptosystem should be secure even when the cipher design is known to the attacker. The only thing that should be hidden is the value of the secret key. Therefore, it is important to make two assumptions: First, it is assumed that the attacker has access to cryptograms between the sender and receiver. Second, the attacker knows about the encryption algorithm, except for the secret key. The proposed cipher does not use the traditional way of encryption, where participants agree on a particular encryption technique and share the secret key using some protocol, but the dynamic encryption concept is used here. The dynamic encryption [5] concept was originally designed by Lars R. Knudsen in 2015, where for each session, the sender use different ciphers from a large pool to encrypt the data. In such situations, it is hard for the attacker to break the system, or it is difficult to make an attack. In such a scenario, the receiver also does not know about the encryption algorithm. The sender sends the compiled encryption algorithm along with the encrypted ciphertext. This approach can be applied to private as well as public-key cryptography. However, such a level of dynamism has a major disadvantage in IoT based ciphers, creating an extra burden for IoT devices. The dynamic encryption concept is useful for standard encryption algorithms that are mainly suitable for desktop/server environments with significant computational power. Therefore, instead of sending a new cipher every time, these IoT devices use the same cipher. The dynamism concept is used in a different sense by only varying the cipher specifications in each new transmission instead of the whole cipher, e.g., changing the number of rounds, switching operations used in cipher, etc. Resource-constrained devices require adaptability to the dynamic change of encryption or decryption components based on resource availabilities, and therefore the dynamism concept can be used here with a different dimension. In BRISK, a dynamism concept is used where not only the key changes its value, but the encryption algorithm also changes its components in each communication and, therefore, extra security is provided to the system.

## 2. Background

### 2.1. RFID

**Figure 1:**
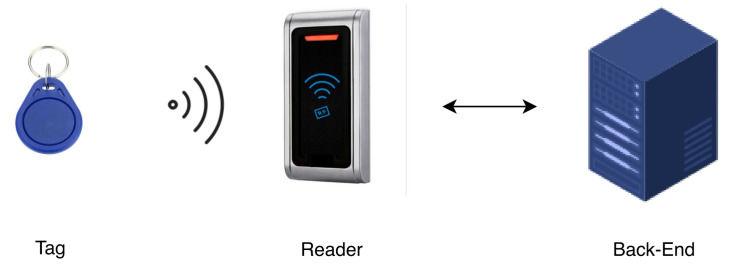
RFID components.

An RFID system mainly consists of three components: readers, tags and a back-end system (see Figure 1). A tag may be attached to any object, and RFID readers are used to identify that object uniquely. The tags may contain any type of information about the object, such as model number, serial number, or other information or characteristics that uniquely define that object. An RFID tag (also known as a transponder) is a small device whose information can be contactlessly read by RFID readers. It contains two parts, namely: an onboard antenna and internal circuitry.

There are several types of tags available (see Table 1) based on frequency, power source and cost. Tags can be categorised as follows:

- Passive Tags: Passive tags absorb the power from radio waves, and they do not have any battery. The absorbed power by tag is used to power the internal circuit and make communication through the antenna.
- Active Tags: In general, active tags are more expensive due to additional expenses on manufactures. Active tags contain the power source onboard. This power source could be a battery that provides power to the antenna and the internal circuit.
- Semi-Active Tags: Semi-active tags contain a battery to give power to the internal circuit, but this power is not used for communication through the antenna. Such tags retain the advantage of both active and passive tags and remove several disadvantages from both. The power can be saved for a long time.

The radio-frequency identification (RFID) system will soon replace the barcode system completely as it is considered the next generation of a barcode. The tags used in RFID can be divided into two types, read-only tags: information stored in this cannot be changed later, and read-write tags: information stored in this can be read or write, but they are more expensive than read-only tags. RFID has several applications in real life, including passports, electronic IDs, door locks, supply chains, electronic payments, etc. Some of the application is given as follows:

- Healthcare: RFID has several applications in healthcare such as patient tracking, patient monitoring, patient drug compliance, patient monitoring, etc.
- Contact-less Payments: Many companies such as MasterCard, VISA, American Express etc., use RFID technology for contactless payments.
- Passport: Several countries like the United States, Japan, and Norway incorporate RFID tags in passports that store traveller history, photographs and other important information.
- Toll Road Payments: Several highway toll booths use RFID technology where vehicles do not stop on the toll booth but directly passes through an E-Z pass lane, and the toll is automatically deducted.
- Product Tracking: RFID tags are mostly used in tracking the inventory throughout the supply chain. These RFID tags are used as a cost-effective way to track products and used as a substitute for the barcode.

### 2.2. Cryptology

**Figure 2:**
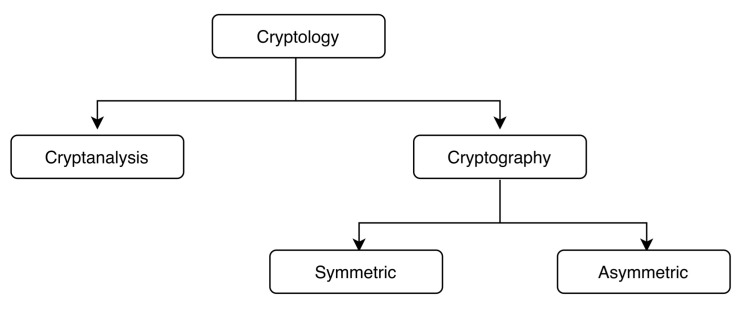
Cryptology.

The more general term *cryptology* is related to encrypting information with the help of encryption algorithms and also analysing these algorithms in terms of security margin. Therefore, in general, the cryptology is divided into two parts: *cryptography* and *cryptanalysis* (see Figure 2). The goal of cryptography is to hide actual information by transforming the information into other non-readable encrypted data. On the other hand, cryptanalysis is more about breaking the cryptosystem. Cryptanalysis is equally important, as without this, we never know how secure our system is.

**Figure 3:**
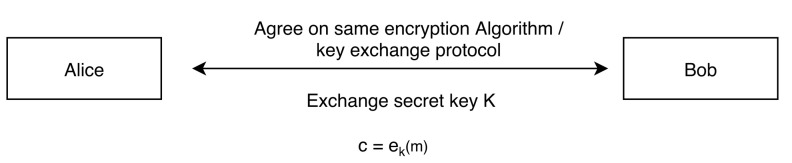
Symmetric key encryption.

Cryptography (Encryption) splits into two parts: *symmetric key encryption* and *asymmetric key encryption*. In symmetric-key encryption (see Figure 3), the sender (Alice) and receiver (Bob) agree on a particular encryption algorithm and key exchange protocol and uses the same key *K* to encrypt or decrypt the data. Key exchange protocol can exchange the key over an insecure channel, and only Alice and Bob know the secret value of the same key. On the other hand, in asymmetric key encryption, every participant has their own key pair called the public and private keys. A public key is known to everyone, and the private key is only known to one participant. Alice can use Bob’s public key to encrypt the data and send it to Bob, and Bob can decrypt the data by using their own private key. However, in this chapter, we use the symmetric key algorithm and therefore, a similar key is used to encrypt and decrypt the data.

### 2.3. Strategy to Design Lightweight Ciphers

The biggest challenge for designers of lightweight algorithms is to make a balance between three things, namely: *performance, security* and *cost*. In block ciphers, the cost-performance trade-off is provided by hardware architecture, the key length of the cipher provides the security-cost trade-off while the number of rounds provides the security-performance trade-off. However, these three properties create a trilemma, and it is only possible to obtain the only pair of goals out of three. In general, there are three basic approaches to design encryption algorithms suitable for lightweight applications as RFID tags:

- Design new ciphers with a goal of low costs for hardware implementation.
- Slightly modify standard algorithms and make it suitable lightweight applications.
- Optimise the implementation cost of standard and trusted algorithms.

One major problem of passive RFID applications is a very small power source. The power consumption for lightweight algorithms should not be more than 15 μA. The following metrics are used to assess the efficiency of any cipher design.

- **Area:** This requirement is generally measured in μm${}^{2}$. To analyse the area requirements of a cipher, it is easy to state the area as gate equivalents (GE). A two-input NAND gate requires the area of one GE, and therefore GE area can be derived by dividing the area in μm${}^{2}$ by area of the two-input NAND gate. Some RFID devices like contactless smart cards or RFID tags require low power consumption and small area, but other factors like throughput are not very important.
- **Cycle:** It represents the number of clock cycles to compute or read the results. A parallel block cipher implementation performs any number of encryption/decryption round operation within one clock cycle. On the other hand, in round-wise implementation, one round function is performed in one cycle.
- **Time:** Time required for a certain operation can be calculated by taking the ratio of cycles and operating frequency and mathematically can be expressed as $t=\frac{cycles}{frequency}$. Time can be expressed in milliseconds [ms].
- **Power** Power consumption is measured in microwatts [μW] and estimated on gate level. The following equation represents the power consumption of *P* in CMOS devices.
  (1) $$P=\left(\frac{1}{2}\cdot C\cdot V_{dd}^{2}+Q_{sc}\cdot V_{dd}\right)\cdot f\cdot N+I_{leak}\cdot V_{dd}$$
  In the above equation, $V_{dd}$ is the supply voltage, *C* is circuit capacitance, $Q_{sc}$ is short circuit charge, *N* is switching activity, $I_{leak}$ is leakage current, and *f* is operating frequency.
- **Throughput:** Throughput is the rate at which output can be produced with respect to time. Throughput is measured in bits per second [bps] and can be calculated when output bits are divided by time. Some RFID reader devices require high throughput because at the same time they read out many devices, while power consumption and the area are not that important for them.
- **Efficiency:** The efficiency is the ratio of throughput and area, and mathematically can be expressed as $efficiency=\frac{throughput}{area}$. This can also be expressed as gate equivalents per bits per second [$\frac{GE}{bps}$].

Another major issue with lightweight applications is low storage. Each cipher has an internal state: cipher state and key state. Ciphers use plaintext to initialise the state, and at this phase, it is called a cipher state while it is modified each round with the action of key, and therefore called key state onwards. The internal state of the cipher is generally stored at each round. In general, for the desktop environment, RAM and ROM are available to store the data, but in the case of RFID tags, it is not possible, and values are stored in registers. Block ciphers generally have a fixed number of rounds, and a large number of rounds requires more memory, as we know that cipher states are saved in the memory after each round. However, in this paper, a dynamic number of rounds concept is used, and therefore, very small applications can also run the block cipher by using a small number of rounds. Table 2 shows the various important cipher components and their values (in terms of area, process and GE) that play an important role in the designing of ciphers.

### 2.4. Lightweight Ciphers

The recently proposed lightweight block ciphers SPECK and SIMON [6], by the National Security Agency(NSA) of the United States of America are two encryption algorithms that are suitable for resource-constrained devices due to small block sizes. SIMON is designed to perform well on hardware devices, while SPECK was designed to perform well on software. Another cipher, Simeck [7], that was designed by combining the good components of SIMON and SPECK. Simeck has very good performance on hardware and has comparable security levels.

Another lightweight block cipher, LEA [8], was designed that has faster performance on software. The experiments performed by designers shows that LEA is faster than AES on ARM, AMD and Intel platforms. LEA is based on simple ARX (modular Addition, bitwise Rotation, and bitwise XOR) operations. These operations are well supported for 32-bit and 64-bit platforms. LEA has a block size of 128-bits with three key sizes 128, 192 and 256 bits. Several other lightweight ciphers have been proposed in the past few years. Some of the well known lightweight block ciphers are: PRESENT [9], TEA [10], KATAN and KTANTAN [11], etc. Several other lightweight ciphers (see Table 3) have become popular in the cryptographic community.

The dynamic encryption [5] concept was introduced in 2015 and has been called “state of the art in cryptology” by Vincent Rijmen, who is one of the designers of AES encryption. Dynamic encryption provides extra security by changing the cryptosystem for each data transfer. Professor Lars Ramkilde Knudsen made the invention at the Technical University of Denmark.

### 2.5. Contribution

In this article, a lightweight block cipher BRISK has been proposed. It supports block sizes of 32-bit, where the block is divided into two of word size 16-bit. BRISK has the following features.

- BRISK is a symmetric block cipher. This implies that the same key is used for encryption as well as decryption.
- BRISK supports two versions of the cipher, and both version use two different types of S-Boxes.
- BRISK is suitable for hardware as well as software. The cipher is word-oriented, which means at a time operations are performed on words of data.
- BRISK follows dynamism in encryption and decryption. For each session of data encryption, it uses either S-Box${}^{1}$ or S-Box${}^{2}$ as a non-linear component. The number of rounds also varies in each session, and therefore it provides better security against the attacker.
- BRISK has a key length of 80-bits that can be divided into 5 subkeys used for the first 5 rounds, and after that, it generates other round keys from the main key.
- BRISK has a very simple design and is very easy to implement. The simple structure also allows easy cryptanalysis of the cipher. It has a low memory requirement, and therefore can be easily implemented on resource-constrained devices with small memory.

The following notations (see Table 4) were used throughput the paper.

## 3. Specifications of BRISK

BRISK is a block cipher from the family of Feistel ciphers. BRISK has a block size of 2n, and each block is divided into two halves of word size *n*, where *n* has the value of 16-bit. So, the cipher takes plaintext input that is divided into two words and produces two-word ciphertext output with the same size. These block sizes are suitable for different applications like the RFID system. The number of rounds depends on the computational power of devices and security requirements. BRISK uses the key length of 80-bit divided into 5 subkeys for initial rounds and then generates new keys from the previous keys using the round function of BRISK. The cipher has both layers of operations that satisfy the confusion and diffusion property of any cipher. For confusion, cipher uses four S-Boxes of size 4-bit, and for diffusion, it uses a permutation layer of 16-bit. The recommended number of rounds for the cipher is 32. However, it can be changed using the dynamism concept and depends on the security requirement of the IoT devices. Based on the hamming weight of the key, cipher chooses which component has to be used. For example, if there are an odd number of 1s in the binary value of key then S-Box${}^{1}$ and P-Box${}^{1}$ is selected while if the number of 1s in the key is even then S-Box${}^{2}$ and P-Box${}^{2}$ is selected to encrypt the data. To derive the key itself, only S-box${}^{1}$ and P-Box${}^{1}$ are used and can be transferred between sender and receiver using the key exchange protocol.

### 3.1. Round Function

**Figure 4:**
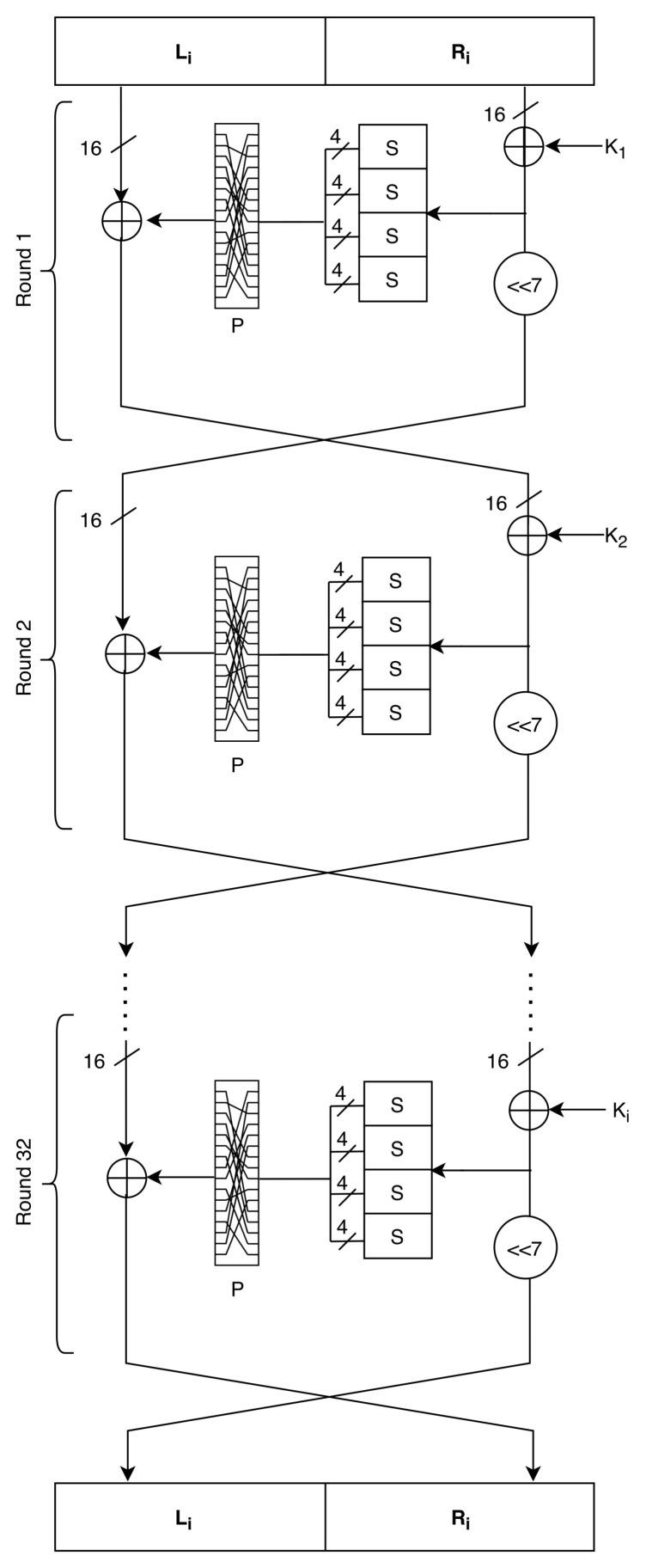
The round function of BRISK.

The algorithm mainly has three components: encryption, decryption and key expansion. The round function of the cipher is shown in Figure 4. The round function of BRISK has the following operations:

- bitwise XOR, ⊕;
- Substitution-Box *S*;
- Permutation-Box *P*.

The round function of BRISK is a map $R_{i}:GF{\left(2\right)}^{n}\times GF{\left(2\right)}^{n}$ that is defined by

(2) $$SL_{i}=\left(SR_{i-1}⊕K_{i}\right)<<7$$

(3) $$SR_{i}=SL_{i-1}⊕P\left(S\left(K_{i}⊕SR_{i-1}\right)\right)$$

where, $k\in GF{\left(2\right)}^{n}$. To decrypt the cipher, the inverse of the round function is used where the substitution box is exchanged with the inverse substitution box, and the permutation box is modified by inverse permutation box. S-Box is one of the major components of any cipher that is used as a non-linearity component. S-Box is the most vulnerable component against linear and differential attacks, and therefore it is important to design a very secure S-Box against these attacks. The S-Boxes used in this cipher have enough security margin against linear and differential attacks. BRISK uses two different S-Boxes (see Table 5 and Table 6) for non-linearity property.

For permutation layer purpose, two different permutations (P-Box${}^{1}$ and P-Box${}^{2}$) are used that permutes 16-bit of word according to the Table 7 and Table 8.

### 3.2. Key Schedule

**Figure 5:**
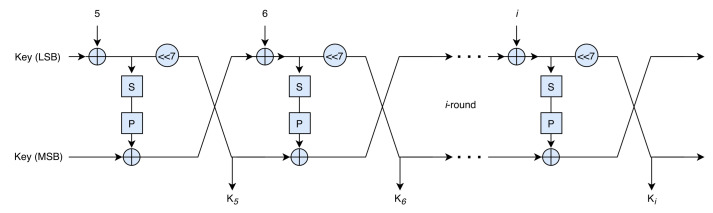
Key generation diagram of BRISK.

The initial key *K* is of size 80-bit and divided into 5 subkeys $k_{0},k_{1},k_{2},k_{3},k_{4}$ where $k_{0}$ is the first (least significant) $\frac{K}{5}$ bits used for first round, $k_{2}$ is next $\frac{K}{5}$ bits for next round and so on. In this way, 5 rounds of cipher get the subkeys from the initial key *K*. To generate the subkeys for next rounds, $k_{3}$ and $k_{4}$ is passed through round function and produce other subkeys. Key scheduling architecture of BRISK is shown in Figure 5.

### 3.3. Key Exchange Protocol

In symmetric-key cryptography, the same key is used to encrypt and decrypt the data on both sides of the communication. This key is generally used only for a session and for the next session key is changed and therefore also called a session key. There are several key exchange algorithms in cryptography such as Elgamal, RSA, Diffie–Hellman, etc. Some of the algorithms are based on integer factorisation such as RSA while others are based on discrete logarithms such as Elgamal or Diffie–Hellman. In this paper, the Diffie–Hellman exchange key algorithm is used with any group for which the discrete logarithmic problem is hard. In elliptic curve cryptography, the points defined on an elliptic curve over a field $Z_{p}$ also form a group, and it uses these points to form a group (instead of integers). Elliptic curve is a set of pairs (points) (x,y) over a field $Z_{p},p>3$ that satisfy the Weierstrass equation $y^{2}≅x^{3}+ax+b\;\mathrm{mod}\;p$ where $a,b\in Z_{p}$ and satisfy the condition $4a^{3}+27b^{2}\neq 0\;\mathrm{mod}\;p$.

Elliptic curve provides very high security with the small size of keys (see Table 9) and is therefore suitable for IoT devices. The security level of 80 means that with exhaustive search, attackers use the computational power of $2^{80}$ computations, and similarly, the security level of 128 means attackers have to use the computational power of $2^{80}$ computation. Instead of taking a normal group, an elliptic curve group is taken and used to define a discrete logarithmic problem for the Diffie–Hellman exchange key algorithm.

**Figure 6:**
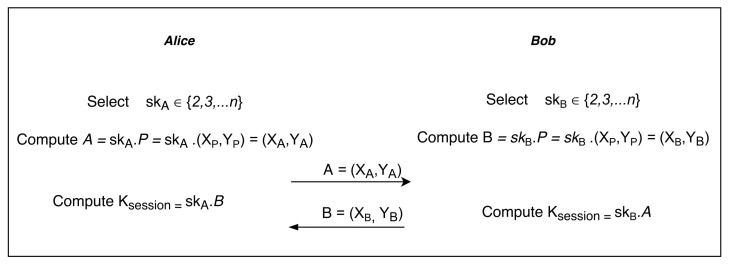
The elliptic curve Diffie–Hellman key exchange (ECDH).

Elliptic curve Diffie–Hellman secret key exchange algorithm is explained with Figure 6. Consider Alice and Bob are exchanging keys with each other over an insecure channel. Alice will choose a number $sk_{A}\in \left(2,\cdots ,n\right)$. These numbers are chosen from the elliptic curve points and private to her. Bob does the same and chose a random number $sk_{B}\in \left(2,\cdots ,n\right)$ from the set of elliptic curve points that is private to him. With the help of a primitive element, Alice and Bob will calculate public parameter *A* and *B* that is transferred to each other. Now, from these two points, Alice and Bob can generate a secret session key $K_{session}$.

## 4. Analysis of the Proposed BRISK Cipher

In this section, the general analysis related to the cryptographic strength of the cipher is illustrated. Two important properties of a secure cipher given by Claude Shannon are: confusion and diffusion. Confusion hides the relationship between key and ciphertext, and diffusion hides the relationship between plaintext and ciphertext. Another important aspect of any cipher is the key length that provides the security level of the cipher.

- To achieve the goal of confusion, BRISK uses non-linear components: S-Box${}^{1}$ and S-Box${}^{2}$.
- The blocks in the cipher are divided into two half words, and both words interchange their sides after each round. Applying this process for several rounds and using permutation boxes P-Box${}^{1}$ and P-Box${}^{2}$ provides the property of diffusion.
- Key length plays an important role in the security of any cipher, and therefore, the cipher uses the key size of 80-bit, which is enough to produce tight security.

Another important aspect is its hardware implementation with a low area on the chip. The goal of this cipher is to find implementation with lower complexity and a simple round function. The area is measured in gate equivalent (GE) and depends on a particular library. However, this cipher is based on dynamic design and the number of rounds or operation changes throughout the implementation and, therefore, instead of writing the exact area, the maximum area is calculated with the highest number of rounds. Note that the user can use a small number of rounds, and in that case, the area will be smaller. The result is mainly based on the ARM standard library for IBM 8RF and 0.13 μm technology. Some basic operations have the following areas: OR (1.25), XOR (2.00), NAND (0.75), NOT (0.75), D flip-flop (4.25), etc. The flip flops are also included, as they are used to store the state and key of the cipher. BRISK has a state of the art implementation size that can also be reduced due to the dynamic concept of the cipher.

The hardware implementation comparison is shown in Table 10.

### Security Evaluation of Cipher

**Figure 7:**
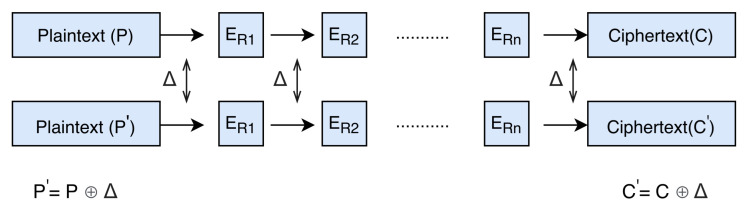
Difference propagation of a plaintext pair.

Differential Cryptanalysis: To analyse the security margin of the cipher, the most powerful attack differential cryptanalysis is applied to the cipher. Differential cryptanalysis was introduced by Biham and Shamir in 1990 for the cryptanalysis of cipher FEAL [39]. This technique was very popular when the full round of Data Encryption Standard (DES) was broken by differential cryptanalysis [40]. The basic idea behind the differential cryptanalysis is to take a pair of plaintexts (*P* and $P^{{}^{\prime }}$) and analyse the propagation of their differences throughout the cipher rounds (see Figure 7).

**Figure 8:**
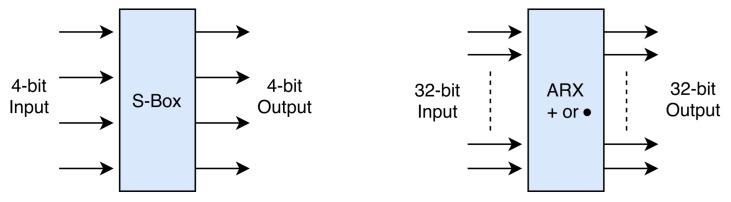
Non-linear components of a cipher.

The sequence of differences through several rounds $E_{R1},E_{R2},\cdots E_{Rn}$ with certain probability *p* is called differential characteristics (also known as trail or path). An ideal cipher should follow random distribution for all input/output pairs and the probability of differential path for the n-bit cipher is $2^{-n}$. If ciphers do not follow the uniform distribution and an attacker can find a certain path with probability ($p>2^{-n}$), then this path can be treated as a distinguisher and can be used to make an attack. To apply differential cryptanalysis, the attacker needs to care about the non-linear component of the cipher. The differences throughout the cipher pass through these non-linear components (S-box or modular addition) with a certain probability (see Figure 8).

For an input difference of the non-linear layer, there could be several possible outputs with different probabilities. The attacker needs to find higher probability output. For such calculations, a difference distribution table (DDT) of the non-linear component is required to generate based on the non-linear layer’s specifications. This table shows the probability of each output for a certain input. Generating such a table for S-boxes that has mainly the size of 8- or 4-bit is possible. The size of difference distribution table (see Table A1) for a 8-bit S-box is $2^{16}$ ($2^{8}$ for 4-bit S-box, respectively). However, for ARX-based ciphers (Addition/Rotation/XOR) based on simple operations, the word size of non-linear components is 16- to 64 bit. Therefore, computing the difference distribution table for these lightweight ciphers is infeasible. Such an attack requires a clever heuristic tool that can provide high probability differential paths for the cipher. Dwivedi et al. [41,42] presented an heuristic tool to find differential path for ARX based ciphers. To find differential path for BRISK, a similar algorithm is used.

The cipher is analysed with differential cryptanalysis using the nested tree search method, and the result is presented in Table 11 for both versions of the cipher (S-Box${}^{1}$ and S-Box${}^{2}$). The detailed result and difference distribution tables are presented in Appendix A. Due to the dynamic nature of the algorithm, BRISK uses two different types of non-linear components and therefore analysing BRISK is equivalent to analyse two different ciphers. In this section, both results are presented, and based on that, the user can choose cipher rounds to provide better security.

Linear Cryptanalysis: Linear cryptanalysis [43] is another powerful cryptanalytic tool to analyse any block cipher. The idea behind linear cryptanalysis is to find a linear equation between plaintext and ciphertext that describes the relationship between input and output bits of the cipher. For a secure cipher, such equations hold with the probability of 0.5 (bias ϵ=0). However, if an attacker can find an equation that holds with ϵ≠0, then such an equation can be converted into an attack. Similar to differential cryptanalysis, here also a probability table is required. However, instead of the propagation of differences of two different plaintexts, here propagation of plaintext is required. The probability bias table of S-Box is called Linear Approximation Table. Similar to the difference distribution table, it is also hard to calculate the difference distribution table for large size blocks that are common in ARX ciphers (see Figure 8). The heuristic tool is used to find random bias values from the table and find an optimal linear path for the cipher. The total bias of the cipher can be calculated using Equation (4).

(4) $$\epsilon_{1,2,3...n}=2^{n-1}\prod_{i=0}^{i=n}\epsilon_{i}$$

Cipher is also analysed with linear cryptanalysis using the nested tree search method, and the result is presented in Table 12 for both versions of cipher (S-Box${}^{1}$ and S-Box${}^{2}$). The detailed result and difference distribution tables are presented in Appendix A.

## 5. Conclusions

This paper presents an encryption algorithm that is mainly suitable for resource-constrained devices such as the Internet of Things. The cipher uses the concept of dynamism and uses two different cipher components to encrypt the data. For each session, the number of rounds can also be different. However, at a time (one session), only one cipher is used, and the attacker is not aware of which cipher is exactly used. Due to the dynamism concept, the cipher provides an extra layer of security. The cipher is also safe against the most powerful cryptanalytic tool called linear and differential cryptanalysis. Cryptanalysis results show that the cipher has enough security margin against these two attacks when using the recommended rounds.

## Figures and Tables

**Table 1:** Specification of active and passive Tags.

Specifications	Active Tag	Passive Tags
Price	2–5 Euro	0.1 Euro
Storage	32–70 KB	32-1K bits
Reading Distance	up to 10 cm	up to 3 cm
Security Capabilities	RSA, SHA, 3DES	250-4K gates

**Table 2:** Requirement of several parameters in selected standard cells of the UMCL18G212T3 library.

Standard Cell	GE	Area (μm${}^{2}$)	Process
XOR	2.67	25.805	0.18 μm
OR	1.33	12.902	0.18 μm
NOR	1.00	9.677	0.18 μm
AND	1.33	12.902	0.18 μm
NAND	1.00	9.677	0.18 μm
NOT	0.67	6.451	0.18 μm

**Table 3:** List of ciphers.

Algorithm	Structure	Block Size	Key Size	No. of Rounds
AES [2]	SPN	128	128/192/256	10/12/14
DES [12]	Feistel	64	54	16
3DES [13]	Feistel	64	56/112/168	48
LEA [8]	Feistel	128	128,192,256	24/28/32
RC2 [14]	Feistel	64	8–1024	18
RC5 [15]	Feistel	32/64/128	0–2040	1–255
RC6 [16]	Feistel	128	128/192/256	20
Present [17]	SPN	64	80/128	31
XTEA [18]	Feistel	128	128	64
TEA [10]	Feistel	64	128	64
mCrypton [19]	SPN	64	64/96/128	12
Twofish [20]	Feistel	128	128/192/256	16
Idea [21]	Lai–Massey	64	128	8.5
GOST [22]	Feistel	64	256	32
Katan [11]	Stream	32/48/64	80	254
PRINTcipher [23]	SPN	48/46	48/96	48/96
Blowfish [24]	Feistel	64	32–448	16
Khundra [25]	Feistel	64	80	18
Skipjack [26]	Feistel	64	80	32
Misty1 [27]	Feistel	64	128	8
Prince [28]	SPN	64	128	12
Sea [29]	Feistel	48/96/144	48,96,144	Variable

**Table 4:** Notations used in the paper.

Notation	Description
*n*	half of the block size
$K,k_{i}$	Key and Subkeys
sk	Secret Key
S-Box (S)	Substitution Box
P-Box (P)	Permutation Box
p⋘r	left circular shift of *p* by *r* position
q⋙r	right circular shift of *q* by *r* position
$SL_{i-1}$	left n-bit input words to the *i*-th round
$SR_{i-1}$	right n-bit input words to the *i*-th round

**Table 5:** Substitution layer${}^{1}$ of BRISK.

x	0	1	2	3	4	5	6	7	8	9	A	B	C	D	E	F
S(x)	5	C	B	6	9	0	D	A	E	3	8	F	4	1	7	2

**Table 6:** Substitution layer${}^{2}$ of BRISK.

x	0	1	2	3	4	5	6	7	8	9	A	B	C	D	E	F
S(x)	C	5	6	B	9	0	A	D	3	E	F	8	4	7	1	2

**Table 7:** Permutation layer${}^{1}$ of Agile.

x	0	1	2	3	4	5	6	7	8	9	A	B	C	D	E	F
P(x)	15	10	5	0	3	14	9	4	2	7	13	8	12	1	6	11

**Table 8:** Permutation layer${}^{2}$ of Agile.

x	0	1	2	3	4	5	6	7	8	9	A	B	C	D	E	F
P(x)	15	5	10	0	6	4	9	14	2	7	13	8	12	1	3	11

**Table 9:** Evaluation of security requirement.

Cryptosystem	Algorithm Family	Security Level			
		80	128	192	256
Elliptic Curve Digital Signature Algorithm (ECDSA), Elliptic-curve Diffie–Hellman (ECDH)	Elliptical Curve	160 bit	256 bit	384 bit	512 bit
Digital Signature Algorithm (DSA), Elgamal, Diffie–Hellman (DH)	Discrete logarithm	1024 bit	3072 bit	7680 bit	15,360 bit
Rivest-Shamir-Adleman (RSA)	Integer factorization	1024 bit	3072 bit	7680 bit	15,360 bit

**Table 10:** Lightweight block ciphers for RFID systems.

Algorithm	Key Size	Block Size	Technology (μm)	Area (GE)
CLEFIA [30]	128	128	0.13	2488
LED [31]	128	64	0.13	3194
XTEA [18]	128	64	0.13	2521
KLEIN [32]	64	64	0.13	1432
SEA [33]	96	96	0.13	2562
PRINCE [34]	128	64	0.13	2953
AES-128 [35]	128	128	0.13	3100
NOEKEON [33]	128	128	0.13	2880
PRESENT-80 [17]	80	64	0.13	2195
RECTANGLE [36]	80	64	0.13	1111
Piccolo-80 [37]	80	64	0.13	683
Piccolo-128 [37]	128	64	0.13	758
mCrypton-64 [19]	64	128	0.13	2420
mCrypton-96 [19]	96	128	0.13	2681
mCrypton-128 [19]	128	128	0.13	2949
SIMON [38]	64	32	0.13	562
SIMON [38]	96	48	0.13	796
SIMON [38]	128	64	0.13	1026
SPECK [38]	64	32	0.13	549
SPECK [38]	96	48	0.13	778
SPECK [38]	128	64	0.13	1005
BRISK	80	32	0.13	<580

**Table 11:** Differential trails for BRISK cipher.

Cipher Version	Plaintext	Ciphertext	Bias	Active S-Boxes
S-Box${}^{1}$ and P-Box${}^{1}$	0x8000 0x0000	0x9000 0x5004	30.41	12
S-Box${}^{2}$ and P-Box${}^{2}$	0x0000 0x8000	0x0080 0x000a	30	11

**Table 12:** Linear trails for BRISK cipher.

Cipher Version	Plaintext	Ciphertext	Bias	Active S-Boxes
S-Box${}^{1}$ and P-Box${}^{1}$	0xa43b 0x26c6	0x0001 0x0080	31	17
S-Box${}^{2}$ and P-Box${}^{2}$	0xc189 0xb110	0x2800 0x0240	30.41	17

## Data Availability

Not applicable.

## Appendix A

**Table A1:** Linear approximation table (LAT) of 4-bit S-box${}^{1}$.

I/O	0	1	2	3	4	5	6	7	8	9	A	B	C	D	E	F
**0**	8	0	0	0	0	0	0	0	0	0	0	0	0	0	0	0
**1**	0	−2	2	0	−2	0	0	2	−2	0	0	2	−4	2	−2	−4
**2**	0	0	4	0	0	−4	0	0	2	−2	2	2	2	2	−2	2
**3**	0	2	−2	−4	−2	0	0	−2	0	2	2	0	−2	0	−4	2
**4**	0	0	−2	2	−2	−2	0	4	−2	−2	0	−4	0	0	−2	2
**5**	0	2	0	−2	4	2	0	2	0	−2	4	−2	0	2	0	-2
**6**	0	0	−2	−2	−2	2	−4	0	0	−4	−2	2	2	2	0	0
**7**	0	−2	0	−2	−4	2	4	2	2	0	2	0	2	0	2	0
**8**	0	0	2	−2	0	0	−2	2	−2	2	0	0	−2	2	4	4
**9**	0	−6	0	−2	2	0	−2	0	0	2	0	−2	2	0	−2	0
**A**	0	0	2	2	0	4	2	−2	0	0	−2	−2	0	4	−2	2
**B**	0	−2	0	−2	2	0	2	0	2	−4	−2	0	−4	−2	0	2
**C**	0	0	0	0	−2	−2	−2	−2	4	0	0	−4	−2	2	2	−2
**D**	0	−2	−2	4	0	2	−2	0	2	0	4	2	−2	0	0	2
**E**	0	0	4	0	−2	2	−2	−2	−2	−2	2	−2	0	−4	0	0
**F**	0	2	2	0	0	2	−2	4	4	2	−2	0	0	−2	−2	0

**Table A2:** Linear approximation table (LAT) of 4-bit S-box${}^{2}$.

I/O	0	1	2	3	4	5	6	7	8	9	A	B	C	D	E	F
**0**	8	0	0	0	0	0	0	0	0	0	0	0	0	0	0	0
**1**	0	0	2	2	−2	2	−4	0	−4	0	2	−2	−2	−2	0	0
**2**	0	−2	0	2	0	2	0	−2	4	−2	4	2	0	−2	0	2
**3**	0	2	−2	−4	−2	0	0	−2	0	2	2	0	−2	0	−4	2
**4**	0	−2	−2	0	2	0	0	2	0	−2	−2	0	−6	0	0	2
**5**	0	2	0	−2	4	2	0	2	0	−2	4	−2	0	2	0	−2
**6**	0	0	−2	−2	−2	2	−4	0	0	−4	−2	2	2	2	0	0
**7**	0	0	4	−4	0	0	0	0	0	0	0	0	0	0	4	4
**8**	0	−2	−2	0	−4	2	2	4	0	2	2	0	0	2	2	0
**9**	0	−6	0	−2	2	0	−2	0	0	2	0	−2	2	0	−2	0
**A**	0	0	2	2	0	4	2	−2	0	0	−2	−2	0	4	−2	2
**B**	0	0	0	0	−2	−2	2	2	0	−4	0	−4	2	−2	−2	2
**C**	0	0	0	0	−2	−2	−2	−2	4	0	0	−4	−2	2	2	−2
**D**	0	0	2	2	0	−4	−2	2	0	0	2	2	0	4	−2	2
**E**	0	−2	4	−2	−2	0	2	0	0	−2	0	2	−2	0	−2	−4
**F**	0	2	2	0	0	2	−2	4	4	2	−2	0	0	−2	−2	0

**Table A3:** Difference distribution table (DDT) of 4-bit S-box${}^{1}$.

I/O	0	1	2	3	4	5	6	7	8	9	A	B	C	D	E	F
**0**	16	0	0	0	0	0	0	0	0	0	0	0	0	0	0	0
**1**	0	0	0	0	0	4	0	4	0	4	0	0	0	4	0	0
**2**	0	0	0	4	2	0	2	0	0	0	4	0	2	0	2	0
**3**	0	2	0	4	0	0	4	2	0	0	0	2	0	2	0	0
**4**	0	0	2	0	0	0	2	0	0	0	2	0	6	2	0	2
**5**	0	2	0	0	0	4	0	2	2	0	2	2	0	0	0	2
**6**	0	2	2	0	0	0	4	0	2	2	0	0	2	0	2	0
**7**	0	2	0	0	2	0	0	0	0	2	0	4	2	0	0	4
**8**	0	2	0	2	0	0	0	0	2	2	2	2	0	2	0	2
**9**	0	0	2	0	4	0	2	0	2	0	0	0	0	2	2	2
**A**	0	0	2	2	0	4	0	0	0	2	0	2	0	2	2	0
**B**	0	0	0	0	2	0	0	2	4	0	2	2	2	0	2	0
**C**	0	2	0	2	2	4	0	2	0	0	0	0	2	2	0	0
**D**	0	2	4	0	2	0	0	0	2	2	2	0	0	0	2	0
**E**	0	2	2	2	0	0	0	2	0	2	0	0	0	0	2	4
**F**	0	0	2	0	2	0	2	2	2	0	2	2	0	0	2	0

**Table A4:** Difference distribution table (DDT) of 4-bit S-Box${}^{2}$.

I/O	0	1	2	3	4	5	6	7	8	9	A	B	C	D	E	F
**0**	16	0	0	0	0	0	0	0	0	0	0	0	0	0	0	0
**1**	0	2	2	2	0	2	0	0	0	2	0	0	0	2	2	2
**2**	0	2	4	2	0	0	0	0	2	0	2	0	0	0	4	0
**3**	0	2	0	4	0	0	2	0	0	0	0	2	2	4	0	0
**4**	0	0	2	0	2	0	2	2	2	0	2	2	0	0	2	0
**5**	0	0	0	0	0	6	0	2	2	0	2	4	0	0	0	0
**6**	0	2	0	0	2	0	4	0	4	2	0	0	2	0	0	0
**7**	0	0	0	0	0	0	0	0	2	4	2	4	0	2	0	2
**8**	0	4	0	2	2	0	0	0	0	0	0	2	2	0	0	4
**9**	0	0	4	0	2	0	2	0	0	0	0	0	0	2	4	2
**A**	0	2	2	2	0	2	0	0	0	2	0	0	0	2	2	2
**B**	0	0	0	0	0	0	0	0	2	0	2	0	8	2	0	2
**C**	0	2	0	2	2	4	2	4	0	0	0	0	0	0	0	0
**D**	0	0	0	2	4	0	2	0	0	2	4	0	2	0	0	0
**E**	0	0	0	0	0	2	0	6	0	4	0	0	0	2	0	2
**F**	0	0	2	0	2	0	2	2	2	0	2	2	0	0	2	0

**Table A5:** Linear trails for BRISK cipher with S-Box${}^{1}$ and P-Box${}^{1}$.

Round	Block1	Block2	Bias	Active S-Box
1	0xa43b	0x26c6	8	4
2	0x6313	0x0be7	6	3
3	0xf385	0xe200	3	2
4	0x0071	0x00c0	2	1
5	0x6000	0x0200	1	1
6	0x0001	0x0080	2	1
7	0x4000	0x0010	2	1
8	0x0800	0x4000	2	1
9	0x0020	0x0040	2	1
10	0x2000	0x0200	1	1
11	0x0001	0x0080	1	1
Total Probability:			31	17

**Table A6:** Differential trails for BRISK cipher with S-Box${}^{1}$ and P-Box${}^{1}$.

Round	Block1	Block2	$\log_{2}p$	Active S-Box
1	0x8000	0x0000	0	0
2	0x0000	0x8000	3	1
3	0x8000	0x1000	2	1
4	0x1000	0x9800	6	2
5	0x9800	0x1006	5	2
6	0x1006	0x0040	3	1
7	0x0040	0x5006	5	2
8	0x5006	0x9000	3	1
9	0x9000	0x5004	3.41	2
Total Probability:			30.41	12

**Table A7:** Linear trails for BRISK cipher with S-Box${}^{2}$ and P-Box${}^{2}$.

Round	Block1	Block2	Bias	Active S-Box
1	0xc189	0xb110	5	3
2	0x8858	0x0918	5	3
3	0x8c04	0xe400	4	2
4	0x0072	0x000e	1	1
5	0x0700	0x0020	2	1
6	0x1000	0x0040	2	1
7	0x2000	0x0010	2	1
8	0x0800	0x0010	2	1
9	0x0800	0x0050	2	1
10	0x2800	0x0050	2	1
11	0x2800	0x0240	2.41	2
Total Probability:			30.41	17

**Table A8:** Differential trails for BRISK cipher with S-Box${}^{2}$ and P-Box${}^{2}$.

Round	Block1	Block2	$\log_{2}p$	Active S-Box
1	0x0000	0x8000	2	1
2	0x0040	0x0001	3	1
3	0x0080	0x1040	6	2
4	0x2008	0x0020	2	1
5	0x1000	0x0008	2	1
6	0x0400	0x0000	0	0
7	0x0000	0x0400	3	1
8	0x0002	0x0008	3	1
9	0x0400	0x1000	3	1
10	0x0008	0x0001	3	1
11	0x0080	0x000a	3	1
Total Probability:			30	11
